# It could be a ‘Golden Goose’: a qualitative study of views in primary care on an emergency admission risk prediction tool prior to implementation

**DOI:** 10.1186/s12875-015-0398-3

**Published:** 2016-01-06

**Authors:** Alison Porter, Mark Rhys Kingston, Bridie Angela Evans, Hayley Hutchings, Shirley Whitman, Helen Snooks

**Affiliations:** Swansea University Medical School, ILS2, Swansea, SA2 8PP UK; SUCCESS Service User group, Swansea University Medical School, ILS2, Swansea, SA2 8PP UK

**Keywords:** Normalisation process theory, Process evaluation, Intervention studies, Primary care, Clinical prediction rule, Risk assessment, Health services/utilisation, Emergency admissions, Chronic disease

## Abstract

**Background:**

Rising demand for health care has prompted interest in new technologies to support a shift of care from hospital to community and primary care, which may require clinicians to undertake new working practices. A predictive risk stratification tool (Prism) was developed for use in primary care to estimate patients’ risk of an emergency hospital admission. As part of an evaluation of Prism, we aimed to understand what might be needed to bring Prism into effective use by exploring clinicians and practice managers’ attitudes and expectations about using it. We were informed by Normalisation Process Theory (NPT) which examines the work needed to bring an innovation into use.

**Methods:**

We conducted 4 focus groups and 10 interviews with a total of 43 primary care doctors and colleagues from 32 general practices. All were recorded and transcribed. Analysis focussed in particular on the construct of ‘coherence’ within NPT, which examines how people understand an innovation and its purpose.

**Results:**

Respondents were in agreement that Prism was a technological formalisation of existing practice, and that it would function as a support to clinical judgment, rather than replacing it. There was broad consensus about the role it might have in delivering new models of care based on active management, but there were doubts about the scope for making a difference to some patients and about whether Prism could identify at-risk patients not already known to the clinical team. Respondents did not expect using the tool to be onerous, but were concerned about the work which might follow in delivering care. Any potential value would not be of the tool in isolation, but would depend on the availability of support services.

**Conclusions:**

Policy imperatives and the pressure of rising demand meant respondents were open to trying out Prism, despite underlying uncertainty about what difference it could make.

**Trial registration:**

Controlled Clinical Trials no. ISRCTN55538212.

## Background

Rising demand for health care, particularly from people living with long term conditions, is prompting service providers and policy makers to turn to new technologies in order to improve efficiency and effectiveness. Clinical risk prediction models use demographic information, diagnoses, and service use data to stratify a population’s risk of having or developing a specified disease, or experiencing an outcome such as emergency admission to hospital [1, 2]. In combination with a software platform, the models provide clinical risk prediction tools [3], whose two functions in supporting health care are case finding for improved clinical management, and resource allocation through budgeting or service planning [4]. In terms of case finding, such tools are seen as potentially more accurate and consistent than clinical opinion in identifying patients at risk of unscheduled hospital admission [5]. In recent years, contractual mechanisms in all four nations of the UK have incentivised risk profiling and stratification as part of a proactive model of care for patients at risk of emergency admission [6, 7]. The funding commitment is considerable: £160 million per year in England alone [6].

Though a systematic review identified 27 validated risk models for emergency hospital admissions in use internationally [8], their clinical and cost effectiveness have not been demonstrated and practical aspects of adoption and use in general practice are poorly understood [9]. A range of risk prediction tools—including the Combined Model [10] developed for NHS England, and QAdmissions [11]—are intended to produce stratified lists of patients, but there is some variation in the underlying modelling (for example, whether or not secondary care data is used alongside primary care data). Prism [12] is a tool developed specifically for NHS Wales, using a 37-variable model based on Welsh data sets, and updated monthly with data from GP practices, and designed to reflect the particular service configuration of the Welsh NHS and the service needs of the Welsh population. We are currently evaluating Prism in general practice in Wales [13], including examining how Prism is being implemented into practice.

Implementation into health care of new technology such as risk prediction tools can be slow and difficult [14, 15]. There is an increasing acknowledgement of the complexities of the process of bringing new technology into routine use: what is implemented is a package of behaviour change, not just a piece of equipment or protocol [16]; it is a process over time [16, 17] and it involves a complex social system, in which multiple parties are involved [17–19], some of which may be resistant [20].

Normalisation Process Theory (NPT) [21] is increasingly being used as a conceptual framework to examine and explain implementation of innovations in health care [22]. NPT emphasises the nature of implementation as a process which entails sustained work by the parties involved, and suggests that four constructs help us understand how innovation is brought into normal everyday practice: how people understand the innovation and its purpose (coherence); what decisions are taken about using it, based on perceived advantages (cognitive participation); what people do to bring the innovation into everyday use (collective action); how an innovation is reviewed, modified or abandoned (reflexive monitoring) [23].

Our qualitative study aims to understand what might be needed to bring Prism into effective use by exploring clinicians’ and practice managers’ attitudes and expectations about using it. We used NPT to inform our analysis, focussing in particular on the first construct, coherence. Our study contributes new understanding of the ambiguities of the real-world expectations of primary care practitioners when presented with an opportunity to adopt a technology into practice. It will both help to inform effective implementation, and to bring new insights to process evaluation of interventions which are being trialled to assess their impact.

## Methods

### Setting and intervention

We undertook this qualitative study of the process of implementing the Prism risk prediction tool in primary care as part of a progressive cluster randomised trial of the tool, in line with recent MRC recommendations on process evaluation [24]. PRISMATIC (ISRCTN55538212) (Prism Risk Stratification Model: A Trial in Chronic conditions management) aims to estimate Prism’s effects on delivery of care, patient satisfaction, quality of life and resources used [13]. PRISMATIC is under way in one Welsh health board area where 32 practices volunteered to participate. The intervention consisted of Prism software, installed on the practice computer system and providing a range of searchable graphical and numerical presentations of risk information at individual or practice level, automatically updated monthly from routine data. To support this, practices were also provided with a training session of approximately 1 h, delivered in the practice; clinical support through two locally appointed ‘GP champions’; a telephone ‘help desk’ at the NHS Wales Informatics Service (NWIS), during working hours; and a user-friendly handbook of guidance on using Prism. Participating practices received a payment of £1250 to cover costs associated with taking part in the trial.

Qualitative data collection took place between October 2012 and April 2013, after the Prism software was installed on the practice computer system, but before Prism was activated for use or training provided. During the course of the data collection, NHS Wales announced that the contract with primary care providers for 2013–14 would include an additional payment for active care management of at least 0.5 % of the practice list predicted to be at high risk of hospital admission [7]. Some data collection took place before this announcement, and some after it.

### Participants

We invited each GP who had been nominated as lead for Prism in participating practices to attend one of four focus groups, along with other staff members (such as practice manager or nurse) if desired. 33 respondents attended focus groups—Group A (5 GPs; 4 practice managers; 1 nurse), Group B (4:3:1), Group C (7:1:0), Group D (5:2:0). We also interviewed 10 GPs who were unable to attend, by telephone (*n* = 8) or in person (*n* = 2), giving a total of 43 participants from across all 32 practices. Participants represented a mix of urban, rural and semi-rural practices. 21 were female. Mean number of years of experience in their role was 14 (range: 3 to 28).

### Data collection

We devised a topic guide (piloted before use) for focus groups and interviews, covering knowledge of existing predictive risk tools; approaches currently used to identify patients at risk of hospitalisation; motivations for participating in the PRISMATIC trial; expectations of Prism use and impact; and any concerns. During the course of the focus group/interview, we presented a summary of the Prism tool with screenshots to enable respondents to understand and visualise how they might use it in practice. Focus groups, conducted by BAE or AP supported by MK, lasted 65–90 min. Interviews, conducted by the same researchers, lasted 28–40 min. All were recorded, with consent, and fully transcribed, except for one interview where detailed notes were taken.

### Analysis

We used May and Finch’s [21] NPT to frame our analysis. We focused on the first of their constructs, coherence, to examine what understanding participating GPs and primary care colleagues had of Prism before they started to use the innovation. In line with NPT, we considered coherence in terms of various tasks which people (individually or collectively) may undertake in relation to an innovation:

1. differentiating it from other practices;
2. agreeing its purpose;
3. understanding how they might use it;
4. identifying its potential value.

NPT suggests that each of these tasks is shaped by factors that promote or inhibit the extent to which participants look on a new practice as meaningful.

In line with the method outlined by Bradley et al., our analysis integrated inductive and deductive approaches [25]. We developed an initial coding frame for analysis, informed by NPT and by the major concepts identified by BAE, AP, MK and service user SW during an initial review of transcripts, and revised and refined this frame during analysis. We used NVIVO 9 software to organise and manage data, to code into groups and connect data into meaningful themes. AP and MK led coding, comparing results and meeting with all authors to confirm consistency and resolve discrepancies through discussion and comparison with raw data. To strengthen credibility, we presented findings to three GPs associated with the study who agreed they were ‘recognisable’ and seemed to ‘fit’ with their experience [26].

### Ethics

The PRISMATIC trial, including this qualitative component, received approval from the National Research Ethics Service for Wales (10/MRE09/25). We gained informed consent from all participants before data collection. We followed recommended ethical standards for conducting this study and storing all data [27].

## Results

### Defining and differentiating Prism from current practice

Respondents discussed what they understood Prism to be, and how it offered something distinct from existing practice. The consensus was that Prism was a potentially useful technological contribution to case-finding as part of the current trend in general practice towards identifying individuals for case management, through a more proactive, rather than reactive, model of care:

*We want a systematic way rather than just seeing people opportunistically because they are ill, we want to get there before they’re ill and on the point of going into hospital. We want to do it in a more orderly manner.* [GP 30, Group D]

The Prism tool was not seen as something entirely new. Many respondents made comparison between the principles and process of Prism and condition-specific risk prediction tools with which they were already familiar, such as the Wells score (estimating the probability of deep vein thrombosis or pulmonary embolism) and Framingham score (cardiovascular risk).

Views varied on whether the Prism technology would be more accurate than the GPs’ own judgement. While they strongly defended their clinical expertise and patient knowledge, some respondents recognised that a formal and systematic process could complement current clinical practice, including the ‘*intuitive risk assessments’* [GP 30, Group D] they routinely carried out. Though colleagues such as practice managers were seen as having a role in accessing the data, most respondents suggested that risk scores needed to be interpreted by GPs, informed by their knowledge of individual patient situations. As a minimum, information from the risk prediction technology would confirm their opinions; at best, it could enhance clinicians’ role in case finding, supplementing their clinical and local expertise by centralising information, giving reminders and highlighting the unusual. It was also observed that the tool could provide a technological rationalisation or justification for clinical decision-making:

*We can always refer back to that tool that “this is the scoring system, that was the reason I admitted this patient or they did not need admission”.* [GP 25, Group B]

It was also suggested by one respondent that the process of implementing the tool could in itself be a spur to change in practice:

*We have been thinking for a while about looking more in depth at some of the—certain individuals. And I think this might give us the impetus to actually carry that through.* [GP 15, Interview]

### Agreeing a purpose for Prism

Many respondents described the potential of Prism in supporting policy imperatives to prevent admissions—‘*something that we’re very much being pressured to do’* [GP 16, IV]—while at the same time enabling practices to meet contractual and reporting responsibilities. Prism was seen as being able to help with the identification of patients at high-risk of emergency admission, so that home based nursing care could be provided through a community support team:

*That’s how I would go about it, you see, identifying the patients that may have a problem, put in place, or mobilise forces that might be able to go in, and help a little bit—you know, if somebody has a chest infection and they’re having difficulty in breathing, they’re in need of—intravenous antibiotics, the [community support] team can do that at home—she doesn’t have to go in.* [GP 9, Interview]

However, a range of reservations were expressed by many of the respondents. One was that, while the tool might be able to identify patients at risk, its purpose would be limited in that it would not necessarily be telling practice staff anything they did not already know:

*I suppose, one of the overwhelming senses, was that if somebody were to give us a list of our top 100 patients, we’d say, “Well, yeah, you know, we already know about them.” ....We felt that the chance of this churning out unexpected patients was probably pretty low.* [GP 15, Interview]

The second reservation was that, though patients may be identified as high risk, it was not necessarily possible to do anything to mitigate that risk:

*The idea is that you can predict who they are and you can do something about it, so they don’t need to be going into hospital. And that sort of assumes lots of things, really. I mean, it assumes first of all that you can predict it, but secondly it presumes that some of these things are modifiable.* [GP 15, Interview]

There were, among a minority of respondents, anxieties about implications in terms of performance management, if practices were to identify needs but then fail to respond to them:

*So, if your patient has a certain level of risk, in the top 5 %…and you haven’t [been able] to get them lower…that’s my worry, because it’ll be like a stick to beat you.* [GP 12, Group C]

Though the function of Prism was generally identified as being individual case finding, a minority of respondents recognised a potential role in service planning. They saw Prism as an opportunity to demonstrate how to improve community services by highlighting patients’ unmet needs:

*If this helps us define and gives evidence to what we need, that’s where I see it coming from.* [GP 13, Group C]

In interviews and focus groups, most respondents reflected on Prism from an individual perspective, rather than reporting any collective viewpoint in their practice. However, a minority of respondents did report on discussions with colleagues about Prism, with GPs saying that they had persuaded partners, and some practice managers that they had convinced GPs, to take the opportunity to try the technology.

### Understanding how they might use Prism

Respondents described the work that they thought would be required of them, or their practice colleagues, to bring Prism into use. From the majority, there was a sense of open-mindedness about trying it, with some uncertainty about what work it would actually involve. One GP speculated that in technical terms it would be relatively straightforward to install and use:

*If it looks like that on the webpage, then that’s very usable, isn’t it. It’s clicking and it’s very organised and the figures are there nice and neatly and you can scroll up and down over a cup of coffee.* [GP 19, Group D]

A minority of respondents described plans for working together with other practice staff to bring Prism into use:

*We’ll involve the whole practice. We’ll involve the nurses—the clinical side, isn’t it? And we’ll have a look at it in clinical meetings, which we have in any case, and we’ll go from there, I think.* [GP 1, Interview]

*It wouldn’t be everybody using it, but I think as long as we’ve got a doctor onboard…I’m there, and then it’s the nurse—a small group…Sometimes if you get too many people involved, nothing gets done.* [Practice Manager 7, Group A]

*I think obviously a meeting first of all…to lead us in the right direction…[work with the patient] will come my way.* [Practice Nurse, Group B]

However, more often there was a sense that there was no coherent interest in Prism across the practice, with some individuals being more inclined to use it than others. There was an acknowledgement that it might not be easy to persuade colleagues to use Prism, because it could be seen as bringing additional work, in a context where clinicians already felt fully stretched:

*If we say, “Well, we’re gonna do this, but it might make X hours extra work,” [my partners] will say to me, “Well, where’s that X hours gonna come from?”.* [GP 9, Interview]

*We struggle at the moment anyway—everybody’s the same with time, with clinics, scripts, mail—everything. After all of this there’s not much time to put in.* [Practice Manager 4, Group C].

As well as concerns about the work involved in implementing Prism (learning how to use it, logging on, running searches) there were some concerns about the additional work which might come from needing to actively case manage any patients identified:

*If you are presented with a list of patients who are deemed to be high risk and there may be people that you haven’t had contact with, that obviously is gonna generate work because you can’t really ignore the fact that they’re deemed at risk and you’d then have to visit or contact them*.[GP 25, Group C]

*Especially if this tool identified patients that are unknown to us, then you’ve gotta go digging…Because we know—we all know Dai down the road, with COPD, who’s back and forth all the time, because he’s in the surgery all the time. But, then, Mr Thomas down here, who we said, “Well, who the hell is he?”.* [Practice Manager 31, Group A]

### Identifying potential value for Prism

GPs and practice staff showed a willingness and open-mindedness about trying the Prism risk prediction tool. Some were curious, keen to find out about new technology or to avoid missing extra resources or information which could improve their patient care. Most saw an opportunity to help their general practice by supplementing their access to data and supporting the move towards proactive health care. But underlying the apparent curiosity, there was an urgent and anxious search for ways to change working practices and pressures, to move away from current practices which were seen as unsustainable, a case of ‘*trying to keep our head above water.’* [GP 18, Group B]

Despite concerns that the risk prediction technology could add to their workload, many felt they had to try it because the alternative was not an option for them.

*I’ll give it a go, see if it does work, if it doesn’t at least we tried, otherwise we just stay in the status quo, which we can’t stay in as the current health service is being funded.* [GP 20, Group C]

One GP summed up the mixture of curiosity, uncertainty, and desire for change which prompted them to sign up for the technology:

*So part of me coming [to the focus group] is to see what we can do with it…we all do it [identifying people at risk] on a sort of informal basis. We recognise who’s ill and who’s the really worrying patients…if you can use that [Prism], then it has a benefit but what that is, is a Golden Goose so to speak.* [GP 6, Group D]

Respondents’ willingness to use Prism was in the face of real anxieties about its role in improving care. There was consensus among respondents that Prism’s value was not as a tool in isolation, but as a way of identifying people for referral to community services or resources. If these were not available, then the scope for Prism to make a difference would be limited. Respondents felt challenged by limited capacity, within the practice and among community staff, to respond to identified needs:

*We have to have a system in place, where there is an alternative…if there’s no alternative, then all of our GPs, all we’re gonna do is just say “admit, admit, admit”.* [GP 11, Interview]

Respondents pointed out gaps in the provision of community services, the heavy workloads of district nurses, and the lack of integration between social and voluntary services. Potential negative impacts of Prism were also noted. Respondents expressed anxieties about raising patient expectations inappropriately:

*You know, there’s nothing worse than saying to somebody, “Well, actually, you need this, but we can’t give it to you.” [GP 26, Interview]*

Respondents were also concerned about the potential impact on staff of identifying needs which they were then unable to meet:

*And it may identify work—a workload—or it may identify needs, that we may feel unable to address. That can be demoralising, can’t it?…And frustrating.* [GP 9, Interview]

Despite deep anxieties about extra work and lack of resources, and uncertainties about potential benefits, there was a common sense among respondents that they had to take up an opportunity which had the potential to be beneficial:

*GPs will generally use anything that they feel is of benefit to their patients. I think the difficulty I would have is not really knowing if it is gonna be of benefit. I’m quite happy to give it a go.* [GP 21, Group C]

## Discussion

New health care technologies are promoted as a way to improve the quality of patient care and effectiveness and efficiency of health services [28]. However, uptake and use of new technologies can be slow, even when clinical effectiveness is already known [14, 29]. NPT provides a useful sensitising theory to inform analysis of implementation, identifying coherence—the mechanism by which participants understand an innovation and its purpose—as one of four essential components to the work of bringing an innovation into routine practice. Informed by this, we explored attitudes of general practice staff before they began to pilot the Prism tool. We were interested to know how respondents understood what Prism was and what it was for.

NPT suggests that achieving the embedding of innovation into routine use is dependent on coherence [21], yet previous applications of the theory have identified that achieving coherence can be the most common obstacle to implementation [30–32].

We found that, in terms of the four tasks which May and Finch [21] suggested make up coherence, there was a mixed story from our respondents. There seemed to be clear agreement on what the tool was—a technological formalisation of existing practice—and a common understanding that it would function as a support to clinical judgment, rather than a replacement for it. Respondents generally saw Prism as something additional to their existing workload, rather than at the core of it. Though Prism stratifies the whole patient population, respondents tended to focus in their discussion on those at the highest risk. When it came to agreeing a purpose, there was broad consensus about the role Prism might have in delivering new models of care based on active management of patients, but also some scepticism. Scepticism about the potential impact of Prism went along two contradictory lines: one was that it would not be useful in identifying high-risk patients, because these were already known; the other was that it would uncover previously unknown high risk patients for whom no practical support could be delivered.

In terms of the work needed to put Prism into use, the majority of respondents did not anticipate that gathering information from the tool would be a major task in itself. The anticipated workload came with what followed—since the tool has no scope to make an impact in isolation, it would lead to a new workload in terms of reviewing patient needs, care planning, and identifying and setting up support services. There was some anxiety about inappropriately raised expectations, and, to a small extent, liability for responding to identified needs. These concerns spilled over into the consideration of the potential value of Prism—because respondents were well aware of the constrained supply of community based care, which would limit the scope for delivering care to patients identified through using Prism, and created uncertainty in what Prism could achieve. One respondent’s comment about the ‘Golden Goose’ referenced, deliberately or not, the world of fairytales.^1^ Our findings seem to concur with previous work on case management models—which include identification of patients at risk as one of their core tasks—which has found impact to be limited for many reasons, including difficulty of engaging with GPs [33].

Although we focussed on the NPT component of coherence, there were clear references to other components of the theory—for example, the comments about respondents’ plans to use the tool in clinical meetings relates to the NPT component of collective action. The potential for overlap between constructs, and the influence they can have on each other in the dynamic process of implementation, has previously been noted [22]; we intend to return to the story of Prism implementation to see how the four constructs have played out over time.

What was striking was, on balance, how the pressures of a rising numbers of patients with long term conditions, together with national, regional and local policy drivers to anticipate patient needs and reducing demand for unscheduled and emergency care, meant that they were open to trying out Prism as it offered at least a hope of improvement. Open-minded willingness to try the tool seemed to over-ride the scepticism, anxiety and uncertainty. A stronger and clearer message to practitioners about the potential benefits of the tool would have been a useful support, but the challenge to offering this was the absence of evidence—which is what the PRISMATIC trial itself was designed to produce.

During the course of the fieldwork, changes to the GP contract introduced new rewards for identifying high risk patients, and Prism was well placed to support practices’ response to this. However, what was encouraged by the new payment scheme was identification of patients at highest risk of emergency admission, and interventions for only a small proportion of these. This was a very narrow focus compared with the much wider range of functions and possibilities available through Prism, and one which is at odds with a literature which suggests that the biggest scope for impact is in identifying and working with the groups of patients in the risk band below the very top, whose needs may be less immediate or extreme [34].

### Strengths and limitations of the study

We interviewed 43 primary care staff with a wide range of experiences, from 32 general practices across rural and urban, prosperous and deprived communities. The data provide a comprehensive spread of views in terms of practices and roles represented. However, in most cases we talked to only one person from each practice and so were not able to explore in full the extent to which those people’s views were representative of their colleagues, nor to discuss in any depth the relative merits of using the tool in an individual or group setting. It may be that there were wide ranges in attitude and expectations among colleagues within a single practice.

These practices had volunteered to take part in the PRISMATIC trial, committing themselves to using the tool for a fixed time period, and receiving a small payment to acknowledge the additional demands on time of practice staff associated with participation in the study. Their views may not be typical of doctors and other staff in practices which did not opt to take part in the study.

This paper examines coherence among the potential front-line users. Their views may differ from those of local commissioners and managers with responsibility for services required by patients at risk of emergency admission and likely to be identified by the technology.

During the course of our fieldwork, practices were informed that a new payment would be available for identifying and managing patients at high risk of hospital admission. In those interviews which took place after this announcement, responses would have been informed and potentially influenced by awareness of this payment and the potential role of Prism in earning it, while in the focus groups and interviews before the announcement, respondents would not necessarily have known that such a payment innovation was planned.

## Conclusions

NPT provided a useful framework to help us to examine attitudes of practice staff to a predictive risk tool at the point of its introduction. We know that stages of adoption and implementation are part of a process that comprises both formal organisational decisions and informal decisions by individual users [35]. Overall, respondents’ views were not a coherent story but a mix of open-minded willingness, scepticism, anxiety and uncertainty. This mix of views was observed within individual GPs’, nurses’ and practice managers’ responses, as well as being reflected in a range of viewpoints from different individuals. The fact that Prism was offered to practices as part of a trial meant that it could be perceived as a low risk, ‘no obligation’ option. The small scale incentives which came with participation in the Prismatic trial were simply for taking part, and did not depend on successfully identifying new patients or delivering interventions to them. Taking the first step of agreeing to take part in the trial and installing Prism in the practice required a relatively small investment of time and effort, and—unlike the use of many other risk prediction tools—no cost.

Further research will explore how expectations at the pre-adoption stage compare to decisions and actual use of the technology in general practice. Our PRISMATIC study will follow up general practice staff over 18 months to explore whether and how they use the new technology, allowing us to consider the other mechanisms identified in NPT.

## Footnotes

1 Grimm’s Fairy Tales contain two stories about geese and gold: the goose who lays the golden eggs is the source of undreamt of bounty; but another story has a golden goose to which the unwary stick when they grab it, with unfortunate consequences. We assume that our respondent was thinking of the former.
