# Erratum to: It could be a ‘Golden Goose’: a qualitative study of views in primary care on an emergency admission risk prediction tool prior to implementation

**DOI:** 10.1186/s12875-016-0416-0

**Published:** 2016-02-03

**Authors:** Alison Porter, Mark Rhys Kingston, Bridie Angela Evans, Hayley Hutchings, Shirley Whitman, Helen Snooks

**Affiliations:** Swansea University Medical School, ILS2, Swansea, SA2 8PP, UK; SUCCESS Service User group, Swansea University Medical School, ILS2, Swansea, SA28PP, UK

Unfortunately, the original version of this article [1] did not include an acknowledgements section for the funding information. The Acknowledgements have been included in full in this erratum.
